# Mechanism of action of antimicrobial peptide P5 truncations against *Pseudomonas aeruginosa* and *Staphylococcus aureus*

**DOI:** 10.1186/s13568-019-0843-0

**Published:** 2019-07-30

**Authors:** Ju Young Kwon, Min Kyung Kim, Loredana Mereuta, Chang Ho Seo, Tudor Luchian, Yoonkyung Park

**Affiliations:** 1 Department of Biomedical Science, Chosun University, Gwangju, 61452 South Korea; 2 Department of Physics, Alexandru I. Cuza University, Iasi, Romania; 3 Department of Bioinformatics, Kongju National University, Kongju, 314-701 South Korea; 4 Research Center for Proteineous Materials, Chosun University, Kwangju, 61452 South Korea

**Keywords:** Antimicrobial peptide, Truncated peptide, Anti-biofilm, Mechanism of action

## Abstract

Rates of microbial drug resistance are increasing worldwide; therefore, antimicrobial peptides (AMPs) are considered promising alternative therapeutic agents to antibiotics. AMPs are essential components of the innate immune system and exhibit broad-spectrum antimicrobial activity. P5 is a Cecropin A-Magainin 2 hybrid analog peptide with antimicrobial activity against Gram-negative and Gram-positive bacteria. In the present study, truncated peptides were designed to reduction length, retainment their antimicrobial activity and low toxicity at high concentrations compared with that of the parent peptide P5. The truncated peptides P5-CT1 and P5-NT1 exhibited antibacterial activities against both Gram-negative and Gram-positive bacteria. In contrast, P5-CT2, P5-CT3, P5-NT2, and P5-NT3 showed higher antibacterial activities against gram-positive bacteria compared to Gram-negative bacteria at low concentration of peptides. The truncated peptides showed lower hemolytic activity and toxic effects against mammalian cells compared with those of the parent peptide P5. The levels of several truncated peptides were maintained in the presence of physiological concentrations of salts, indicating their high stability. The results of flow cytometry, propidium iodide uptake, *n*-phenyl-1-naphthylamine uptake, and 3,3′-dipropylthiadicarbocyanine iodide assays showed that these truncated peptides killed microbial cells by increasing membrane permeability, thereby causing membrane damage. The results suggested that truncated peptides of P5 have good potential for use as novel antimicrobial agents.

## Introduction

Antimicrobial peptides (AMPs) are small molecules, 12–50 amino acids in length, and have been isolated from a variety of organisms, including animals, plants, bacteria, insects, and reptiles (Lehrer and Ganz 1999; Nawrot et al. 2014; van Hoek 2014). AMPs are secreted by, and are important for, the innate immune system (Devine and Hancock 2002; Hancock and Sahl 2006; Mendez-Samperio 2013); therefore, they have been used against a broad spectrum of invading pathogenic agents, such as bacteria, viruses, and fungi (Radek and Gallo 2007). Additionally, AMPs show anticancer activity towards stomach cancer, lung carcinoma, acute T cell leukemia (Park et al. 2003), and colon cancer (Cho et al. 2018). Generally, AMPs contain α-helical, β-sheet, and random coil secondary structures, which are necessary to form pores in bacterial membranes (Takahashi et al. 2010; Nguyen et al. 2011). Moreover, the net charge of AMPs is typically positive because of the presence of arginines and lysines (Brown and Hancock 2006; Jenssen et al. 2006), while the net charge of the bacterial membrane is negative. Therefore, AMPs are attracted to, and can disrupt, the bacterial membrane.

P5 is a Cecropin A-Magainin 2 (CA-MA, KWKLFKKIGIGKFLHSAKKF-NH_2_) hybrid analog peptide, and is a cationic 18-amino acid AMP. Cecropin A (CA) is a cationic 37-amino acid AMP that was isolated from *Hyalophora cecropia* pupae (Steiner 1982; Steiner et al. 2009). Magainin 2 (MA) is a cationic 23-amino acid AMP that was isolated from the skin of the African clawed frog, *Xenopus laevis* (Zasloff 1987). Cecropin A and Magainin 2 show high antimicrobial activities and no toxicity toward normal mammalian cells and red blood cells. A CA-MA hybrid peptide was constructed that combined residues 1 to 8 of CA and residues 1 to 12 of MA. The CA-MA hybrid peptide had high antimicrobial activity against bacteria and fungi (Oh et al. 2000; Park et al. 2003, 2006; Ryu et al. 2015). P5 (KWKKLLKKPLLKKLLKKL-NH_2_) was designed to have a high net positive charge and hydrophobicity by a flexible region (Gly-Ile-Gly → P) substitution and Lys (positions 4, 8, 14, 15) and Leu (positions 5, 6, 12, 13, 16, 17, 20) substitutions (Park et al. 2003). P5 showed high antimicrobial activity against Gram-negative and Gram-positive bacteria and fungi, and anticancer activity in stomach cancer, lung carcinoma, and acute T-cell leukemia at low concentrations. However, P5 causes a low level of hemolysis of red blood cells and cytotoxicity in normal mammalian cells (Park et al. 2003; Ryu et al. 2011).

In the present study, the P5 peptide was truncated by two, four, and six amino acids at the C-terminal or N-terminal ends of the 18-amino acid parent peptide to design six truncated peptides, P5-C-terminus (CT)1, P5-CT2, P5-CT3, P5-N-terminus (NT)1, P5-NT2, and P5-NT3. We designed the P5 truncated peptides to have reduced length while retaining high antimicrobial activity and lower toxicity and hemolysis at high concentrations compared with those of the parent peptide P5. We compared the antibacterial activity, cytotoxicity, hemolysis effects, and anti-biofilm activity of the parent peptide with those of the truncated peptides. Our findings suggested that the P5 truncated peptides P5-CT1 and P5-NT1 could be developed as antibiotic therapeutic agents.

## Materials and methods

### Materials

Lipopolysaccharide (LPS; from *Pseudomonas aeruginosa*), Lipoteichoic acid (LTA; from *Staphylococcus aureus*), 3-(4,5-dimetylthiazol-2-yl)-2,5-diphenyltetrazolium bromide (MTT), dimethyl sulfoxide (DMSO), propidium iodide (PI), *n*-phenyl-1-naphthylamine (NPN), HEPES, and 3,3′-dipropylthiadicarbocyanine iodide (DisC_3_-5) were obtained from Sigma-Aldrich (St Louis, MO, USA).

*Pseudomonas aeruginosa* ATCC 27853 and *Staphylococcus aureus* ATCC 25923 were obtained from the ATCC (American Type Culture Collection, Manassas, VA, USA), and *Acinetobacter baumannii* KCTC 2508 and *Bacillus subtilis* KCTC 2217 were obtained from the KCTC (Korean Collection for Type Cultures, Jeongeup-si, Jeollabuk-do, Korea). Human skin epithelial cells (HaCaT cells) were obtained from ATCC.

### Peptide design

We designed six peptides, P5-CT1, P5-CT2, P5-CT3, P5-NT1, P5-NT2, and P5-NT3, by truncating P5 based on a helical wheel projection and its three-dimensional structure. Amino acid sequence analysis of peptides was conducted using the Mobyle@RPBS bioinformatics portal (http://mobyle.rpbs.univ-paris-diderot.fr/cgi-bin/portal.py#welcome) and the HeliQuest site (http://heliquest.ipmc.cnrs.fr).

### Circular dichroism spectra analysis

Circular dichroism (CD) spectra were measured to investigate the secondary structure of peptides in membrane mimic environments. Each peptide was dissolved at 80 µM in 30 mM sodium dodecyl sulfate (SDS), 50% trifluoroethanol (TFE), or 10 mM sodium phosphate buffer (SP buffer pH 7.2). In addition, the secondary structures of the peptides were measured in the presence of 500 µg/mL LTA and LPS. The CD spectra were measured from 190 to 250 nm using a 1-mm quartz cuvette with a JASCO 810 spectropolarimeter (Jasco, Tokyo, Japan).

### Antimicrobial activity

We detected the antimicrobial activity of peptides against Gram-negative bacteria (*P. aeruginosa* ATCC 27853 and *A. baumannii* KCTC 2508) and Gram-positive bacteria (*S. aureus* ATCC 25923 and *B. subtilis* KCTC 2217). The minimum inhibitory concentrations (MICs) of the parental and truncated peptides were determined using the microbroth dilution method, as previously described (Jorgensen 1993). The bacteria were incubated overnight with shaking at 37 °C. The bacteria were diluted to 2 × 10^5^ colony forming units (CFU)/mL in Mueller–Hinton broth (MHB) media. The peptides were serially diluted in 10 mM sodium phosphate buffer and then 50 µL of the bacteria and 50 µL of the peptide solutions were mixed and incubated for 18 h at 37 °C. The MICs were measured as the absorbance at 600 nm using a Versa Max microplate reader (Molecular Devices, Sunnyvale, CA, USA). The samples were analyzed in triplicate.

### Time-kill kinetics assay

The kinetics of the antibacterial activity of the peptides against *P. aeruginosa* ATCC 27853 and *S. aureus* ATCC 25923 were assessed. Bacteria grown to mid-log phase were diluted to 2 × 10^5^ CFU/mL in MHB media. Peptides at 1× and 2× their MICs were added to the bacteria at 37 °C and aliquots of the bacteria were spread on MHB agar plates. The plates were incubated at 37 °C. After overnight culture, the CFUs of bacteria were counted. The results were expressed as the cell survival percentage.

### Anti-biofilm activity

*Pseudomonas aeruginosa* ATCC 27853 and *S. aureus* ATCC 25923 were cultured in MHB media. Bacteria were diluted in MHB media containing 0.2% glucose at 5 × 10^5^ CFU/mL and the diluted bacteria and peptides were added to a 96-well plate, which was incubated at 37 °C. After 24 h, the supernatant was discarded, and biofilms were fixed with 100% methanol for 15 min. The methanol was removed and the biofilms were stained with 0.1% crystal violet for 30 min, after which the crystal violet was rinsed with distilled water. Next, 200 µL of 95% ethanol was added to each well to dissolve the biofilm. After 1 h, the biofilm inhibition activity was measured as the absorbance at 595 nm using a Versa Max microplate reader.

### Hemolytic activity

To determine the hemolytic activities of the peptides against red blood cells (RBCs) from mice, RBCs were centrifuged at 2000×*g* for 5 min and washed with 1 mL of phosphate-buffered saline solution (PBS) three times. The supernatant was removed and the RBCs were diluted to 8% in PBS. After serial dilution, 60 µL of the RBCs and peptides were added to a 96-well plate and incubated at 37 °C for 1 h. The plate was centrifuged at 1500×*g* for 10 min, 100 µL of the supernatant was transferred to a new plate, and the absorbance was measured at 414 nm (Versa-Max enzyme-linked immunosorbent assay (ELISA) reader). Additionally, 0.1% Triton X-100 and PBS were used as positive and negative controls, respectively. The hemolytic activity percentage (hemolysis rate) was calculated using the following equation (Han et al. 2016):

$$ \begin{aligned} {\text{Hemolysis percentage }}\left( \% \right)\, = & \,\left[ {{{\left( {{\text{peptide solution}}\, - \,{\text{negative control}}} \right)} \mathord{\left/ {\vphantom {{\left( {{\text{peptide solution}}\, - \,{\text{negative control}}} \right)} {\left( {{\text{positive control }}{-}{\text{ PBS}}} \right)}}} \right. \kern-0pt} {\left( {{\text{positive control }}{-}{\text{ PBS}}} \right)}}} \right] \\ & \times \, 100. \\ \end{aligned} $$

### Cell culture and cytotoxicity assay

Human skin epithelial cells (HaCaT cells) were cultured in Dulbecco’s modified Eagle’s medium (DMEM) with 10% fetal bovine serum (FBS) at 37 °C in 5% CO_2_. Cytotoxicity was determined using the MTT assay (Deng et al. 2015). HaCaT cells were plated at 2 × 10^4^ cells/well in 96-well plates. After overnight incubation, the peptides were added at various concentrations for 24 h, and then 5 mg/mL of MTT was added to each well. After 1 h, the supernatant was removed and 100 μL of DMSO was added to each well to dissolve the formazan crystals. Cytotoxicity was measured at an absorbance of 570 nm using a microplate reader (VersaMax). The cell viability percentage (cell viability rate) was calculated using the following equation (Kim et al. 2018):

$$ \begin{aligned} {\text{Cell viability percentage }}\left( \% \right)\, = & \,\left( {\text{peptide treated sample}} \right)\, - \,\left( {\text{no peptide treated control}} \right) \\ & \times \, 100. \\ \end{aligned} $$

### Salt sensitivity

The effects of various cations on the antimicrobial activity of P5 and the truncated peptides were determined against *P. aeruginosa* ATCC 27853 and *S. aureus* ATCC 25923. Bacteria were treated with the peptides in the presence of NaCl (50, 100, and 150 mM), MgCl_2_ (0.5, 1, and 2 mM), and FeCl_3_ (1, 4, and 8 µM). The MIC values were the same as those described above.

### Propidium iodide uptake assay

To assess whether the peptides target the bacterial membrane, we performed a propidium iodide (PI) uptake assay. *P. aeruginosa* ATCC 27853 and *S. aureus* ATCC 25923 were cultured to mid-log phase in MHB media and diluted to an optical density at 600 nm (OD_600_) of 0.27 in MHB media. Bacteria were mixed 20 µg/mL of PI (final concentration: 10 µg/mL) and 50 µL of bacteria were added to each well. Next, 50 µL of peptides at different concentrations was added to each well. PI fluorescence was measured at excitation and emission wavelengths of 580 and 620 nm, respectively.

### Outer membrane permeability assay

Outer membrane (OM) permeability was determined by measuring NPN uptake (Loh et al. 1984). *P. aeruginosa* ATCC 27853 was cultured to mid-log phase in MHB media. The bacteria were washed and resuspended to an OD_600_ of 0.2 in 5 mM HEPES (pH 7.2). Next, 100 µL of the bacteria suspension was mixed with 50 µL of 8 µg/mL NPN (final concentration: 2 µg/mL) and different concentrations of the peptides in a 96-well black plate. NPN fluorescence was measured at excitation and emission wavelengths of 350 and 420 nm, respectively.

### Cytoplasmic membrane depolarization assay

The membrane depolarization activities of the peptides were detected using *P. aeruginosa* ATCC 27853 and *S. aureus* ATCC 25923, together with the membrane potential-sensitive fluorescent dye DisC_3_-5, as previously described (Han et al. 2013). Briefly, the bacteria were cultured to mid-log phase in MHB media and washed with 5 mM HEPES buffer containing 20 mM glucose. Bacteria were resuspended to an OD_600_ of 0.05 in a 5 mM HEPES buffer containing 20 mM glucose and 0.1 M KCl, and then DisC_3_-5 (final concentration: 1 µM) (Kim et al. 2018) was added to each well. After 1 h of incubation, the mixture was left to stand; when the reduction in the fluorescence of the mixture was stable, the peptides were added to each well. The fluorescence was measured at excitation and emission wavelengths of 622 and 670 nm, respectively.

### Flow cytometry

Damage to the bacterial membrane was evaluated using flow cytometry. Briefly, *P. aeruginosa* ATCC 27853 and *S. aureus* ATCC 25923 were grown to mid-log phase in MHB media and then washed three times with PBS. The bacteria were diluted to an OD_600_ of 0.26 in MHB media. The bacteria were treated with 1× MIC and 2× MIC of the peptides and with PI at a final concentration of 10 µg/mL, followed by incubation for 30 min at 37 °C. The bacteria were centrifuged at 13,000×*g* for 5 min and resuspended in 500 µL of PBS. The samples were analyzed using a CytoFLEX flow cytometer (Beckman Coulter, Brea, CA, USA).

### Statistical analysis

All data represented from triplicate individual experiments. The results of hemolysis and cytotoxicity were analyzed using *t*-test. Mean differences at *p < 0.05, **p < 0.01 were considered significant.

## Results

### Peptide design and characterization

**Fig. 1:**
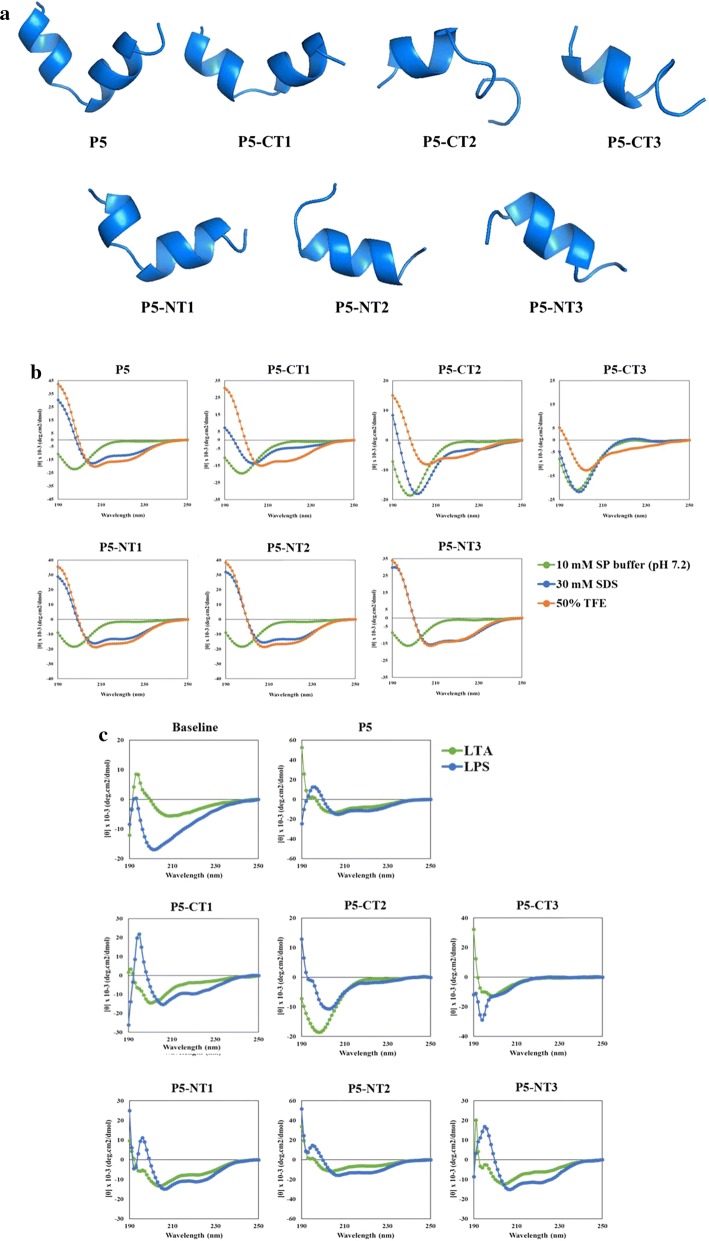
Structural analysis of P5 and its analog peptides. **a** Three-dimensional structure of P5 and its analogue peptides. **b** Circular dichroism (CD) spectra of P5 and its analog peptides were measured in various environments. The peptide concentration was t 80 μM. **c** Interaction of P5 and its analog peptides with in 500 μg/mL lipoteichoic acid (LTA) and lipopolysaccharide (LPS). *CT* C-terminus, *NT* N-terminus, *SP* sodium phosphate, *TFE* trifluoroethanol

The N- and C-terminally truncated peptides of P5 were designed to have a reduced length while retaining their antimicrobial activities. Six truncated peptides were made by truncating two, four, and six amino acids at the C-terminal or N-terminal ends of P5. The net charges of the truncated peptides ranged from + 6 to + 8, and they had lower charges than the parent peptide (Table 1). The three-dimensional conformation of P5 and the truncated peptides are shown in Fig. 1a. P5, P5-CT1, and P5-NT1 had a random coil structure between two α-helix structures. P5-CT2 and P5-CT3 showed a short α-helical structure in the N-terminus and a long random coil structure at the C-terminus. Additionally, P5-NT2 and P5-NT3 had α-helical structures.

**Table 1:** Amino acid sequences and physicochemical parameters of parent and truncation peptides

Peptides	Sequences	Measured MW	Theoretical MW	Net charge	Retention time
P5	KWKKLLKKPLLKKLLKKL-NH_2_	2246.0	2245.4	+ 9	17.425
P5-CT1	KWKKLLKKPLLKKLLK-NH_2_	2004.7	2004.1	+ 8	19.250
P5-CT2	KWKKLLKKPLLKKL-NH_2_	1763.4	1763.0	+ 7	17.925
P5-CT3	KWKKLLKKPLLK-NH_2_	1522.0	1521.5	+ 6	17.075
P5-NT1	KKLLKKPLLKKLLKKL-NH_2_	1931.6	1931.7	+ 8	18.517
P5-NT2	LLKKPLLKKLLKKL-NH_2_	1675.3	1675.3	+ 6	17.825
P5-NT3	KKPLLKKLLKKL-NH_2_	1449.0	1449.1	+ 6	18.067

*MW* molecular weight, *CT* C-terminus, *NT* N-terminus

### Structure of Peptides in Various Environments

To investigate the secondary structure of P5 and the truncated peptides in various membrane mimicking environments (Ma et al. 2015), such as SDS (negatively charged prokaryotic membrane) and TFE (hydrophobic microbial membrane), we obtained CD spectra (Fig. 1b). The CD spectra indicated that P5 and the truncated peptides had a positive peak at 212 nm and negative dichroic band at 195 nm in 10 mM SP buffer (pH 7.2). Therefore, P5 and the truncated peptides had a random coil conformation in 10 mM SP buffer (pH 7.2). P5, P5-CT1, P5-NT1, P5-NT2, and P5-NT3 exhibited a positive peak at 190 nm and two negative dichroic bands at approximately 208 and 222 nm, revealing a α-helical conformation in 30 mM SDS and 50% TFE. In contrast, P5-CT2 and P5-CT3 exhibited random coil conformations in the two solutions, with a positive peak at 212 nm and negative dichroic band at 195 nm. Similarly, P5, P5-CT1, P5-NT1, P5-NT2, and P5-NT3 revealed an α-helical conformation at the presence LTA and LPS, while P5-CT2 and P5-CT3 showed a random coil conformation in both LTA and LPS (Fig. 1c).

### Biological activity of peptides

The antimicrobial activity of P5 and the truncated peptides against Gram-negative bacteria (*P. aeruginosa* ATCC 27853 and *A. baumannii* KCTC 2508) and Gram-positive bacteria (*S. aureus* ATCC 25923 and *B. subtilis* KCTC 2217) were determined using the MIC test (Table 2). The parent peptide showed high antimicrobial activity, with MIC values of 1.56 and 3.13 µM against Gram-negative and Gram-positive bacteria, respectively. Similarly, the antimicrobial activities of P5-CT1 and P5-NT1 were high, with MIC values 1.56 and 3.13 µM, respectively. P5-CT2, P5-CT3, P5-NT2, and P5-NT3 showed low antimicrobial activities against Gram-negative bacteria compared with those against gram-positive bacteria, and exhibited low MIC values against gram-positive bacteria, ranging from 1.56 to 6.25 µM. P5-CT3 had no antimicrobial activity against Gram-negative bacteria.

**Table 2:** Minimum inhibitory concentrations (MICs) of peptides

	MIC (µM)							
	P5	P5-CT1	P5-CT2	P5-CT3	P5-NT1	P5-NT2	P5-NT3	Melittin
Gram-negative bacteria								
*P. aeruginosa* ATCC 27853	3.13	1.56	6.25	> 25	1.56	3.13	6.25	3.13
*A. baumannii* KCTC 2508	1.56	3.13	12.5	> 25	3.13	12.5	25	1.56
Gram-positive bacteria								
*S. aureus* ATCC 25923	1.56	1.56	1.56	3.13	1.56	1.56	1.56	0.78
*B. subtilis* KCTC 2217	1.56	1.56	3.13	6.25	1.56	1.56	1.56	0.78

*CT* C-terminus, *NT* N-terminus

**Fig. 2:**
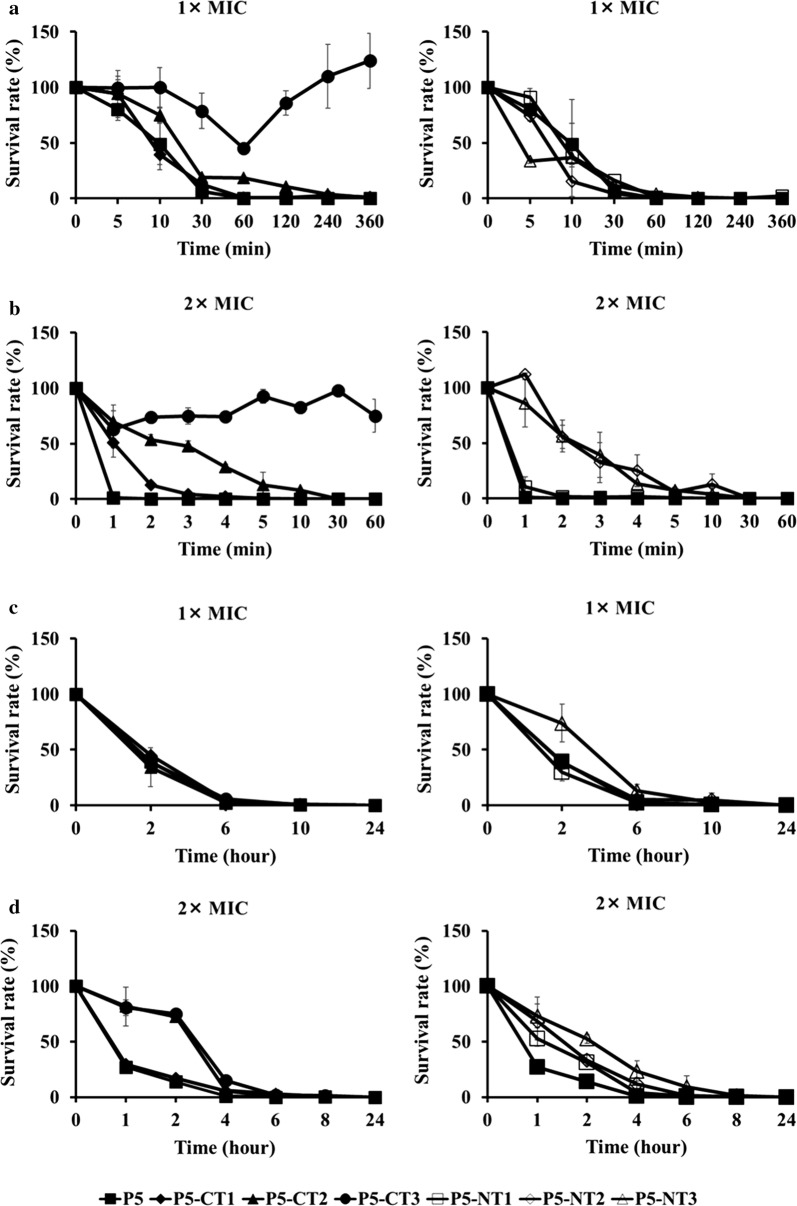
Time-kill kinetic curves of the peptides. Bactericidal kinetics of P5 and its analog peptides were assessed at ×1 MIC and ×2 MIC. **a**, **b** *P. aeruginosa* ATCC 27853 and **c**, **d** *S. aureus* ATCC 25923. *MIC* minimum inhibitory concentration, *CT* C-terminus, *NT* N-terminus

To investigate the antibacterial effect of the peptides, we performed time-kill kinetic experiments (Fig. 2). The time-kill kinetic curves of peptides against *P. aeruginosa* ATCC 27853 and *S. aureus* ATCC 25923 were determined at 1× MIC and 2× MIC. The results showed that killing of *P. aeruginosa* ATCC 27853 occurred more rapidly than that of *S. aureus* ATCC 25923. *P. aeruginosa* ATCC 27853 was killed within 5 min at 2× MIC and within 60 min at 1× MIC after exposure to P5 and the truncated peptides (except for P5-CT3). However, *S. aureus* ATCC 25923 was killed after 6 h at 2× MIC and after 10 h at 1× MIC following the addition P5 and the truncated peptides. The antimicrobial activities of P5 and the truncated peptides were dose- and time-dependent.

**Fig. 3:**
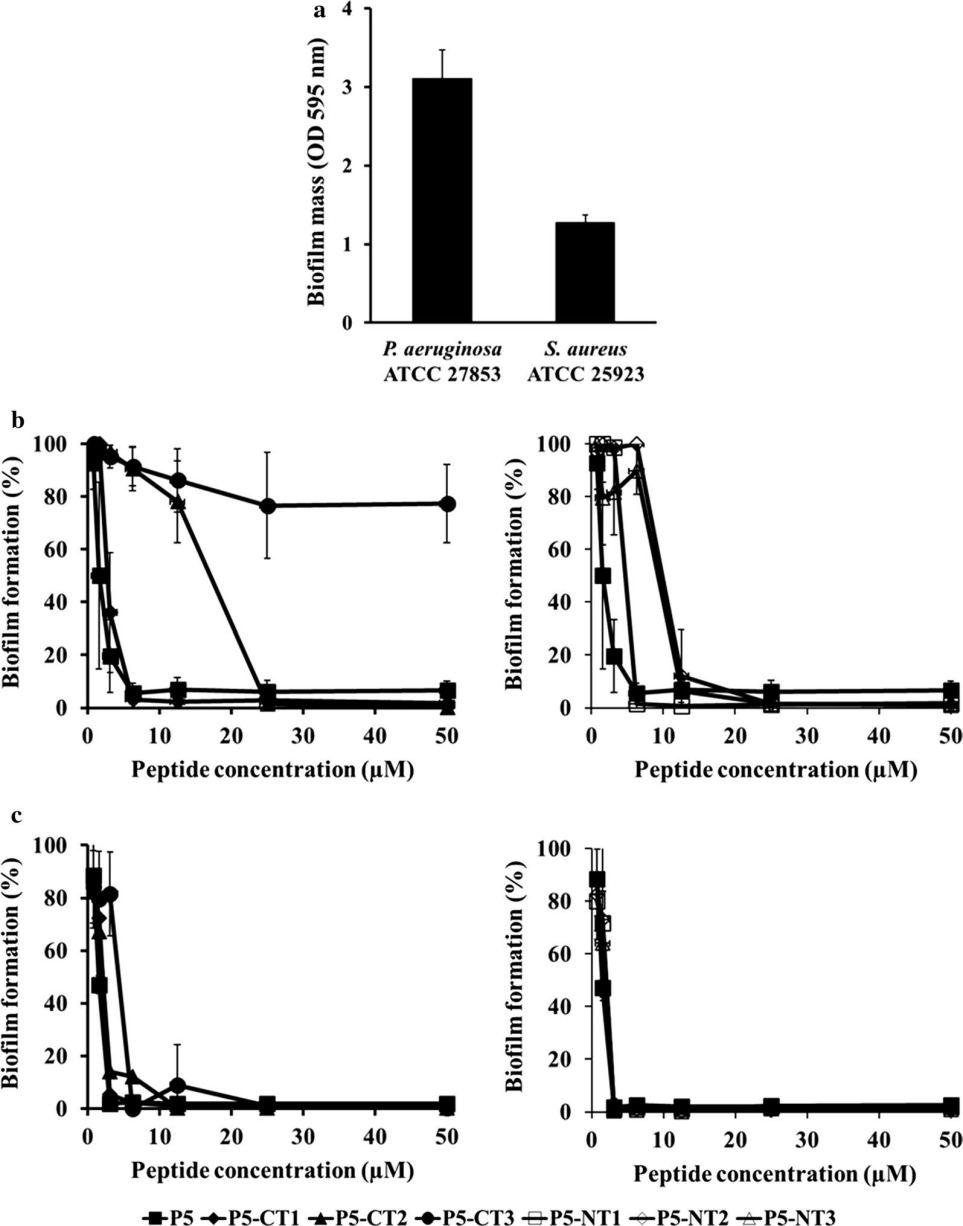
Inhibition of bacterial biofilm formation by the parent peptide and analog peptides against *P. aeruginosa* ATCC 27853 and *S. aureus* ATCC 25923. **a** Biofilm formation by microorganisms detected by crystal violet staining. Anti-biofilm activity of peptides against **b** *P. aeruginosa* ATCC 27853 and **c** *S. aureus* ATCC 25923. *CT* C-terminus, *NT* N-terminus

To assess the anti-biofilm activity of the peptides, we formed biofilms in the presence of P5 and the truncated peptides (Fig. 3). The results showed that biofilm formation by *P. aeruginosa* ATCC 27853 was inhibited at low concentrations of P5 and the truncated peptides; however, P5-CT3 showed no anti-biofilm activity. P5-CT1 and P5-NT1 showed the highest anti-biofilm activities against *P. aeruginosa* ATCC 27853 among all tested peptides. The minimal anti-biofilm inhibitory concentration (MBIC) values of P5 and the truncated peptides (except P5-CT3) against *P. aeruginosa* ATCC 27853 ranged from 6.25 to 25 µM. Additionally, *S. aureus* ATCC 25923 exhibited higher anti-biofilm activity and the MBIC values of P5 and the truncated peptides against *S. aureus* ATCC 25923 ranged from 3.13 to 12.5 µM.

### Hemolytic activity and cytotoxicity of peptides

**Fig. 4:**
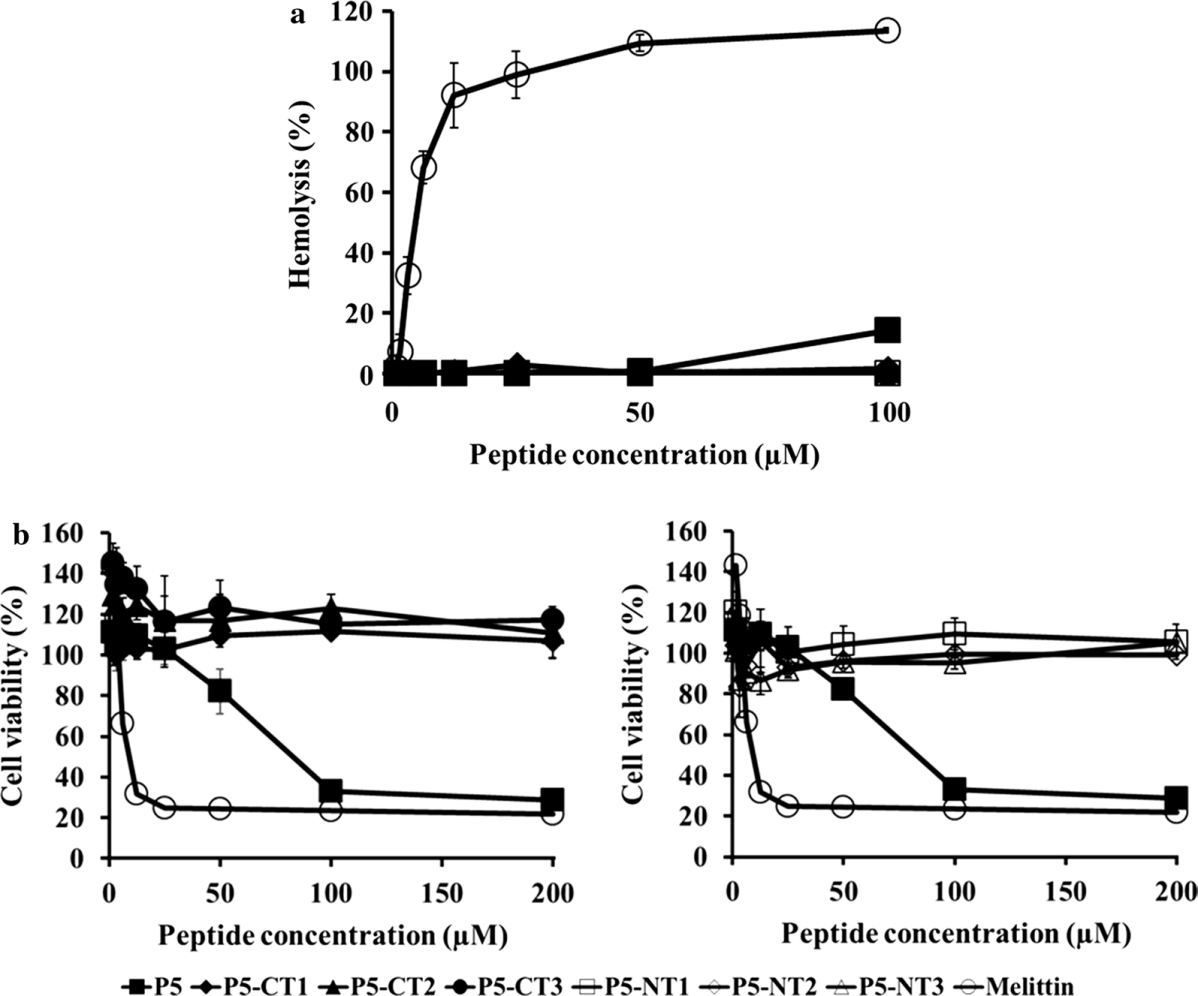
Hemolytic activity of peptides against mouse red blood cells and cytotoxicity of the peptides against HaCaT cells. **a** Hemolytic activity of peptides concentrations from 0 to 100 µM. **b** Cytotoxicity of peptides concentrations from 0 to 200 µM. Each experiment was repeated three times. *CT* C-terminus, *NT* N-terminus

The toxicities of the peptides were determined as the hemolysis of mouse RBCs (Fig. 4a). The hemolytic activities of the peptides are shown in Fig. 4. The parent peptide caused approximately 14% hemolysis at 100 µM, while truncated peptides caused no hemolysis at 100 µM. Melittin from bee venom (Habermann and Jentsch 1967) was used as a positive control (Steiner 1982; Dempsey 1990) and cause approximately 91% hemolysis at 12.5 µM.

The cytotoxicity of P5 and the truncated peptides were assessed against HaCaT cells using the MTT assay (Fig. 4b). The parent peptide was approximately 70% toxic at concentrations over 100 µM, while the truncated peptides showed almost no cytotoxicity at concentrations ranging from 1.56 to 200 µM. Additionally, Melittin, which was used as a positive control, was highly toxic at 1.56 µM.

### Salt sensitivity

The activities of some antimicrobial peptides are affected by salt environments; therefore, the salt sensitivity of peptides was confirmed by adding different salts at physiological concentrations (Table 3). The results revealed the MIC values of the peptides in the presence of salts. The MIC of the parent peptide toward *P. aeruginosa* ATCC 27853 and *S. aureus* ATCC 25923 did not change in different salts at physiological concentrations. The truncated peptides showed stable antimicrobial activity against *P. aeruginosa* ATCC 27853 in the presence Na^+^ (50, 100, 150 mM) and Fe^3+^ (1, 4, 8 µM) Additionally, P5-CT1 and P5-NT1 showed antimicrobial activity in the presence Mg^2+^ (0.5, 1, 2 mM). For *S. aureus* ATCC 25923, the truncated peptides showed stable antimicrobial activity in the presence Na^+^, Mg^2+^, and Fe^3+^ at all concentrations.

**Table 3:** Effect of various concentrations of salt on the antibacterial activity of peptides against *P. aeruginosa* ATCC 27853 and *S. aureus* ATCC 25923

	10 mM SP buffer (pH 7.2)	MIC (µM)								
		NaCl			MgCl_2_			FeCl_3_		
		50 mM	100 mM	150 mM	0.5 mM	1 mM	2 mM	1 µM	4 µM	8 µM
*P. aeruginosa* ATCC 27853										
P5	3.13	3.13	3.13	3.13	3.13	3.13	6.25	3.13	3.13	3.13
P5-CT1	1.56	3.13	3.13	3.13	6.25	6.25	12.5	3.13	3.13	3.13
P5-CT2	6.25	12.5	25	> 25	> 25	> 25	> 25	6.25	6.25	6.25
P5-CT3	> 25	> 25	> 25	> 25	> 25	> 25	> 25	> 25	> 25	> 25
P5-NT1	1.56	3.13	3.13	3.13	6.25	6.25	25	3.13	3.13	3.13
P5-NT2	3.13	6.25	6.25	12.5	12.5	25	> 25	3.13	3.13	3.13
P5-NT3	6.25	6.25	12.5	12.5	12.5	25	> 25	6.25	6.25	6.25
*S. aureus* ATCC 25923										
P5	1.56	1.56	1.56	1.56	1.56	1.56	1.56	1.56	1.56	1.56
P5-CT1	1.56	0.78	0.78	0.78	0.78	0.78	0.78	0.78	0.78	0.78
P5-CT2	1.56	0.78	1.56	1.56	0.78	0.78	1.56	0.78	0.78	0.78
P5-CT3	3.13	3.13	12.5	12.5	3.13	3.13	6.25	1.56	3.13	3.13
P5-NT1	1.56	0.78	0.78	0.78	0.78	0.78	0.78	0.78	0.78	0.78
P5-NT2	1.56	1.56	0.78	0.78	0.78	0.78	1.56	1.56	0.78	1.56
P5-NT3	1.56	0.78	0.78	0.78	0.78	0.78	0.78	0.78	0.78	0.78

*MIC* minimum inhibitory concentration, *CT* C-terminus, *NT* N-terminus

### Outer membrane disruption and permeabilization

**Fig. 5:**
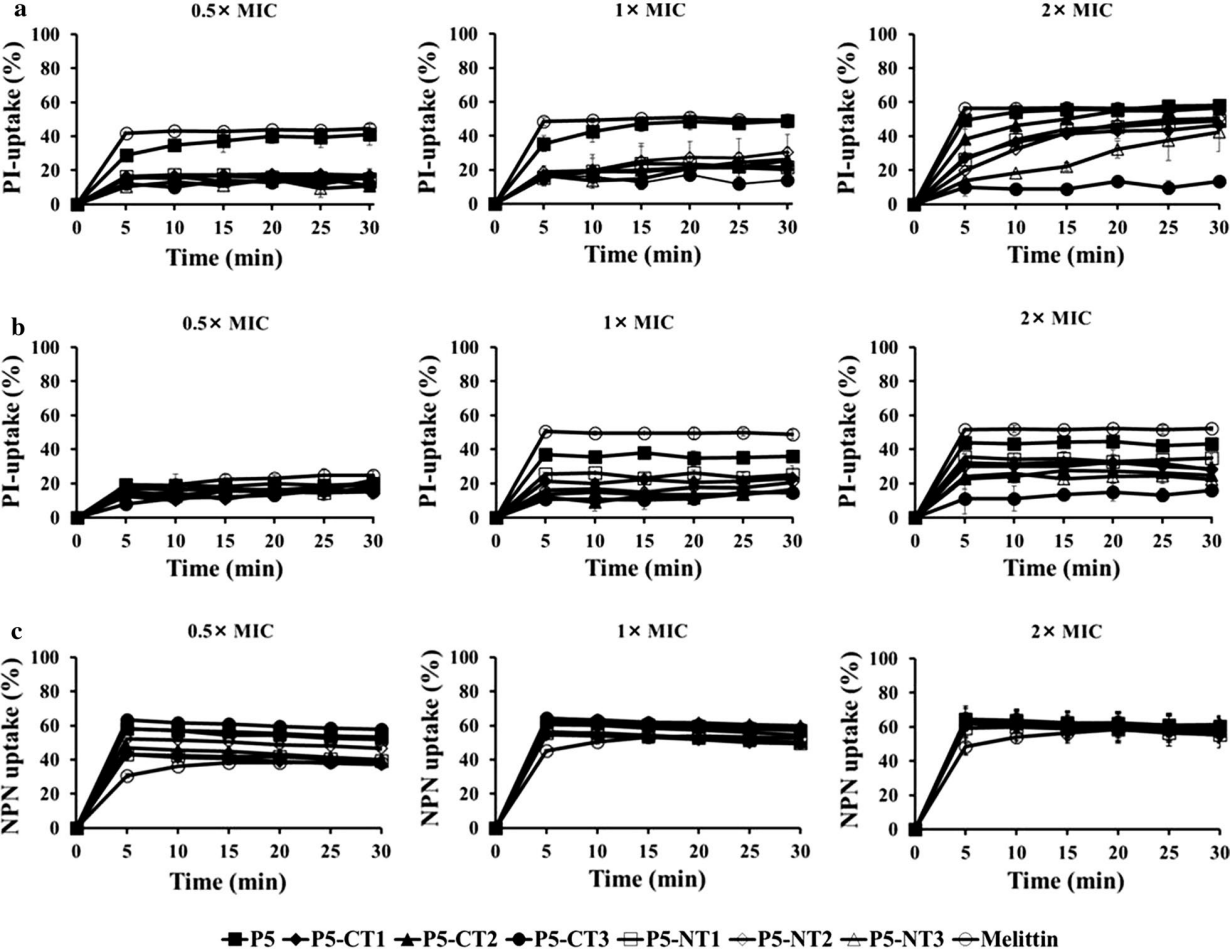
Membrane disruption and permeabilization. Membrane disruption induced by peptides as detected by PI uptake at ×0.5, ×1, and ×2 MIC in **a** *P. aeruginosa* ATCC 27853 and **b** *S. aureus* ATCC 25923. **c** Outer membrane permeabilization induced by peptides as detected using NPN uptake at ×0.5, ×1, and ×2 MIC in *P. aeruginosa* ATCC 27853. *PI* propidium iodide, *MIC* minimum inhibitory concentration, *NPN n*-phenyl-1-naphthylamine, *CT* C-terminus, *NT* N-terminus

We confirmed the membrane disruption and permeabilization by peptides by conducting a PI and NPN uptake assays (Fig. 5). When the bacteria were exposed to P5 or the truncated peptides, the bacterial membrane rapidly became permeable and PI fluorescence increased within 5 min for both *P. aeruginosa* ATCC 27853 and *S. aureus* ATCC 25923 (Fig. 5a, b). Melittin, which is known to cause rapid membrane disruption, also caused a fast increased PI fluorescence. NPN, a hydrophobic probe, was used to investigate permeabilization of the outer membrane of gram-negative bacteria (Fig. 5c). When the outer membrane of the bacteria is damaged, NPN uptake increases (Zhu et al. 2014). P5 and the truncated peptides increased NPN uptake by approximately 50% within 5 min. These results suggested that P5 and the truncated peptides caused membrane disruption to both *P. aeruginosa* ATCC 27853 and *S. aureus* ATCC 25923 and permeabilized the outer membrane of *P. aeruginosa* ATCC 27853.

### Cytoplasmic membrane depolarization

**Fig. 6:**
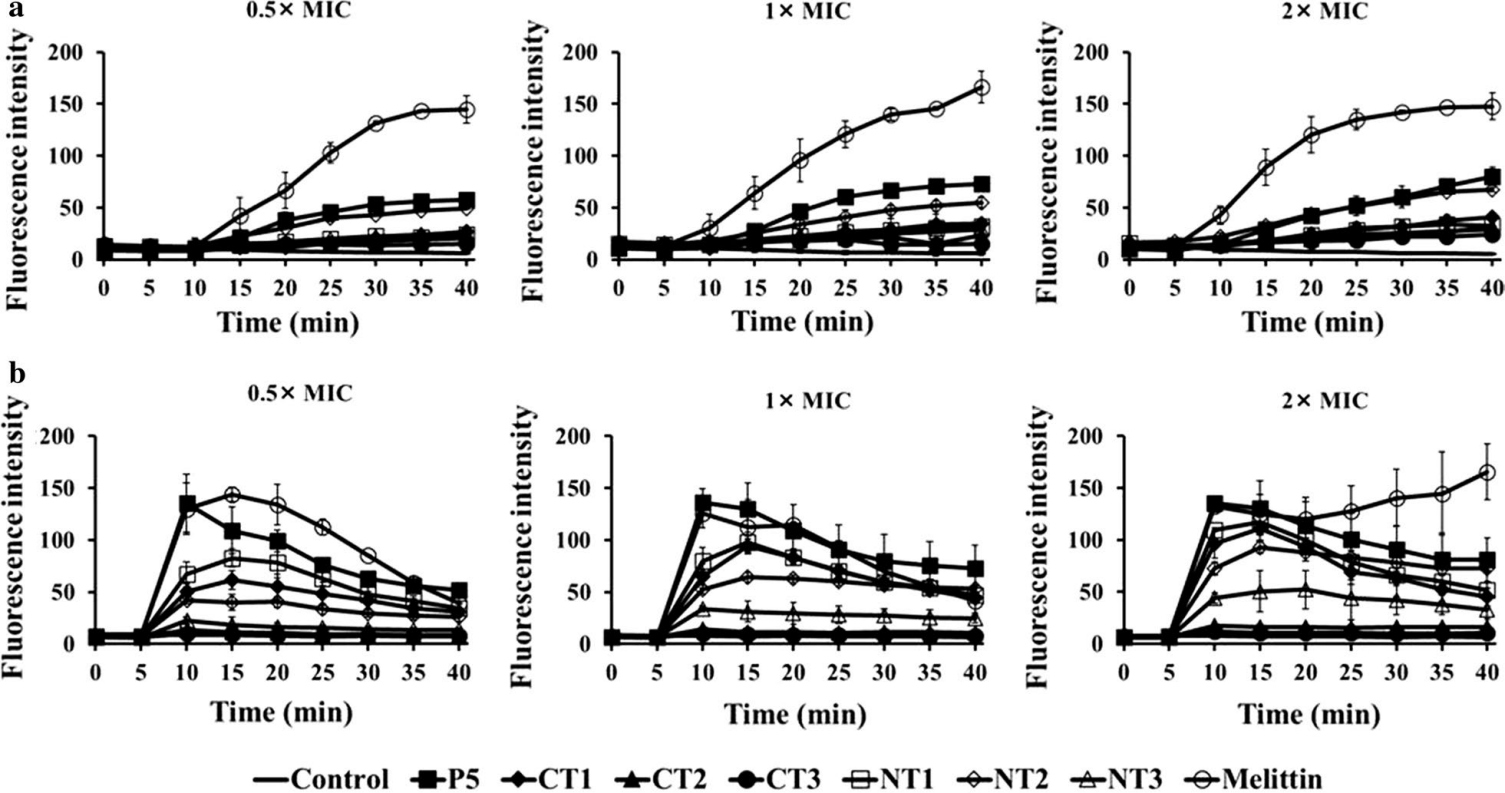
Cytoplasmic membrane depolarization of **a** *P. aeruginosa* ATCC 27853 and **b** *S. aureus* ATCC 25923 was assessed by release of the membrane potential-sensitive dye 3,3′-dipropylthiadicarbocyanine iodide (DisC_3_-5). *MIC* minimum inhibitory concentration, *NPN n*-phenyl-1-naphthylamine, *CT* C-terminus, *NT* N-terminus

DisC_3_-5, a membrane potential-sensitive cationic probe, was used to assess bacterial cytoplasmic membrane depolarization. DisC_3_-5 concentrates in the cytoplasmic membrane and upon membrane disruption, is released into the buffer (Dong et al. 2014) (Fig. 6). The resulting increase in fluorescence can be detected by fluorescence spectrometry. After 5 min of stabilization, the peptides were added to each well and fluorescence was measured for 30 min. The results showed that the fluorescence increased in the presence of P5 and the truncated peptides over time in *P. aeruginosa* ATCC 27853 (Fig. 6a). In *S. aureus* ATCC 25923, except for P5-CT2 and P5-CT3, cytoplasmic membrane depolarization was induced within 5 min by P5 and the truncated peptides (Fig. 6b).

### Flow cytometry analysis

**Fig. 7:**
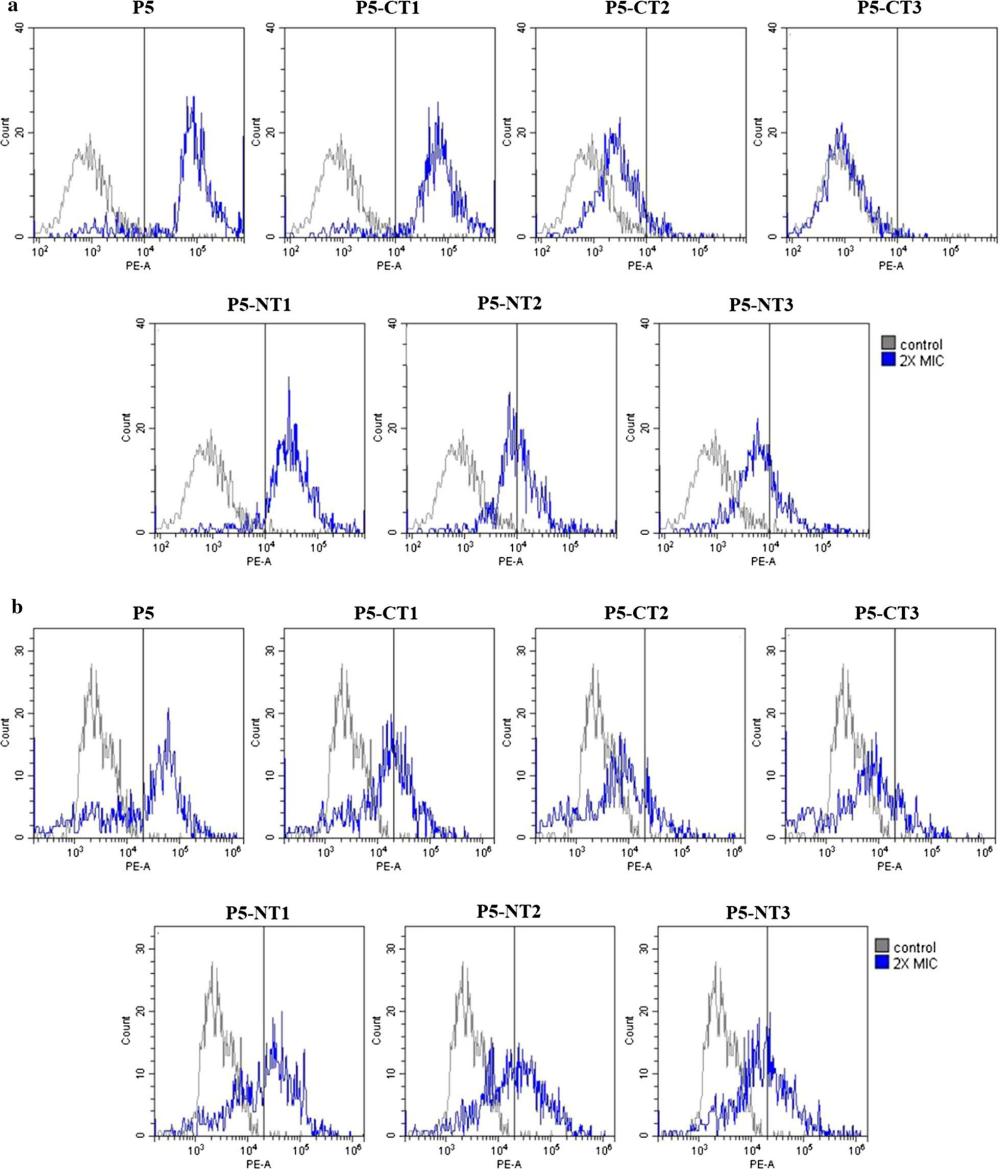
Flow cytometry analysis. Exponential phase *P. aeruginosa* ATCC 27853 and *S. aureus* ATCC 25923 were treated with the peptides, and PI was analyzed by fluorescence activated cell sorting (FACS) flow cytometry. **a** *P. aeruginosa* ATCC 27853, **b** *S. aureus* ATCC 25923. *PI* propidium iodide, *MIC* minimum inhibitory concentration, *NPN n*-phenyl-1-naphthylamine, *CT* C-terminus, *NT* N-terminus

Based on the MIC values of the peptides, their bacterial membrane disruption abilities were measured using fluorescence activated cell sorting (FACS) (Fig. 7). FACS analysis was conducted to count PI-stained bacteria with membranes that were disrupted by the peptides. After 30 min of treatment with 2× the MIC of the peptides, bacterial membrane integrity was measured using FACS. In the control (no peptide) for *P. aeruginosa* ATCC 27853, only 2% PI-positive bacteria were detected, while treatment with P5 produced 85.7% PI-positive bacteria, indicating membrane damage. P5-CT1 stained 86.1% of cells, P5-CT2 stained 9.1% of cells, and P5-CT3 stained 1.2% of cells with PI. P5-NT1, P5-NT2, and P5-NT3 showed values of 87.9%, 46.7%, and 28.5%, respectively. For *S. aureus* ATCC 25923, the control (no peptide) showed 0.9% PI-positive bacteria, while the parent peptide (P5) showed 56.0%. Bacteria treated with P5-CT1, PT-CT2, and P5-CT3 showed 38.9%, 17.6%, and 17.1% PI-staining, respectively. P5-NT1, P5-NT2, and P5-NT3 showed values of 55.0%, 49.0%, and 42.4%, respectively. P5-CT1 and P5-NT1 caused similar levels of membrane disruption to P5, although truncated peptide caused less membrane disruption than P5.

## Discussion

The emergence of antibiotic-resistant bacteria is increasing worldwide; therefore, novel antimicrobial agents are required (Burgess 2014). AMPs, which are essential in the innate immune system, have broad-spectrum antimicrobial activities and can overcome the limitations of traditional antibiotic agents (Pathak and Chauhan 2011; Chen et al. 2013). AMPs show potential as novel therapeutic agents to kill antibiotic-resistant bacteria (Mahlapuu et al. 2016). The primary mechanism of action of AMPs is membrane disruption to induce rapid death of bacteria (Ting et al. 2014). However, developing AMPs for therapeutic applications is difficult, and technological hurdles must be overcome, including the peptides’ stability in various environments, cell selectivity, and reduction in toxicity (Fox et al. 2012; Haney et al. 2012; Liu et al. 2013; Ting et al. 2014). Therefore, designing AMPs with high antimicrobial activity and low cytotoxicity and hemolytic activity would be useful.

In a previous study, P5, a CA-MA analog peptide, was designed to have an increased net positive charge and hydrophobicity by Lys (positions 4, 8, 14, 15) and Leu (positions 5, 6, 12, 13, 16, 17, 20) substitutions, as well as a flexible region (Gly-Ile-Gly → P) substitution. The CA-MA analog peptide P5 parent has stronger antibacterial and antifungal activity, and shows no hemolysis compared with CA-MA (Shin et al. 1998; Park et al. 2003; Mereuta et al. 2014). P5 shows broad-spectrum antimicrobial activity and causes membrane disruption (Park et al. 2003, 2006). Additionally, P5 has low cytotoxicity and hemolytic activity (Park et al. 2003; Ryu et al. 2011).

In the present study, we modified P5 to reduce its length while maintaining its antimicrobial activity. Truncated peptides of P5 were designed such that the 18 amino acids of P5 were truncated at the C-terminus (P5-CT1, P5-CT2, P5-CT3) or N-terminus (P5-NT1, P5-NT2, P5-NT3). The three-dimensional structures of P5-CT1 and P5-NT1, which were truncated by two amino acids, P5-CT2 and P5-NT2, which were truncated by four amino acids, and P5-CT3 and P5-NT3, which were truncated by six amino acids, were determined (Fig. 1). P5-CT1 and P5-NT1 possessed two α-helix structures, while the other truncation peptides had one α-helix structure. In particular, P5-CT1 and P5-NT1 showed structures similar to the parent peptide, and P5-CT2 and P5-CT3 showed a more random coiled conformation than an α-helical conformation. Thus, the truncated peptides of P5 were classified as amphipathic α-helix structure peptides. Additionally, the truncated peptides had lower net positive charges than the parent peptide (Table 1).

Gram-positive bacterial cell walls contain LTA and LPS is an endotoxin found in the outer membrane of Gram-negative bacteria. Cationic amphipathic AMPs can spontaneously insert into the membrane of bacteria, causing disruption (Jamasbi et al. 2014). We measured the peptides’ interaction with LTA and LPS (Bhattacharjya and Ramamoorthy 2009) using CD spectra. Figure 1c showed that P5-CT1, P5-NT1, P5-NT2, and P5-NT3 could bind more LTA and LPS than the parent peptide. The peptides could bind to LTA and LPS, which are present on *S. aureus* and *P. aeruginosa*, to form an α-helix structure; therefore, we expected that the peptides would bind to the bacterial membrane and cause membrane disruption as their primary antimicrobial mechanism.

The P5 truncated peptides were examined to determine their antimicrobial activity against Gram-negative and Gram-positive bacteria (Table 2). Except for P5-CT3, P5 and the truncated peptides showed antimicrobial activity. In particular, the MICs of P5-CT1 and P5-NT1 were similar to those of the parent peptide. The antimicrobial activity of the truncated peptides against Gram-negative bacteria (*P. aeruginosa* ATCC 27853 and *A. baumannii* KCTC 2508) decreased as additional amino acids were removed. P5-CT3, which was truncated by six amino acids, exhibited almost no antimicrobial activity. However, the antimicrobial activity against Gram-negative bacteria decreased when truncation of the C-terminal end was greater than that at the N-terminus. In contrast, for C-terminal truncation, the antimicrobial activity against gram-positive bacteria (*S. aureus* ATCC 25923 and *B. subtilis* KCTC 2217) decreased by more than that of the parent peptide. The antimicrobial activity against Gram-positive bacteria was retained following N-terminal truncation. These results suggested that the C-terminus of P5 is more important than the N-terminus for activity against Gram-negative bacteria, while the C-terminal and N-terminal truncations are equally important for anti-Gram-positive bacteria activity.

Bacterial biofilms are resistant to antibiotic therapy by reducing antibiotic susceptibility and by affecting the host immune systems; therefore, infections involving biofilms are difficult to treat (Bjarnsholt 2013). We investigated the anti-biofilm activity of the peptides. P5-CT1 and P5-NT1 could inhibit biofilm formation at low concentrations, similar to P5 (Fig. 3). We concluded that the P5-CT1 and P5-NT1 showed potential to treat biofilm-related infections of *P. aeruginosa* and *S. aureus*.

Next, we analyzed the hemolytic activities and cytotoxicities of the truncated peptides. The parent peptide showed hemolytic activity at 100 µM and cytotoxicity at 200 µM (Fig. 4). Both P5 and the truncated peptides at their MIC levels exhibited no hemolytic activity or cytotoxicity. These results suggested that the truncated peptides of P5 could be developed as safe therapeutic agents.

To develop AMPs as therapeutic agents, several limiting factors must be overcome. The salt sensitivity of AMPs is a major limiting factor in their development as therapeutic agents (Park et al. 2004). The presence of salt cations can affect the antimicrobial activity of AMPs (Huang et al. 2011). Therefore, we investigated the effects of salts on the antimicrobial activity of truncated peptides at various physiological concentrations (Table 3). The parent peptide was not affected by high salt concentration. None of the truncated peptides were sensitive to salt in terms of their activities against *P. aeruginosa* ATCC 27853 and *S. aureus* ATCC 25923; however, the presence of divalent cations caused some sensitivity against *P. aeruginosa* ATCC 27853. Overall, P5 and the truncated peptides were stable in the presence of salt cations, and thus showed potential as therapeutic agents.

Furthermore, we confirmed the mechanism of action of the P5 truncated peptides through PI uptake, NPN uptake, DisC_3_-5, and FACS assays. All the truncated peptides induced disruption of the outer and inner membranes, and the mechanism of action was the same as that of the parent peptide (Park et al. 2003, 2006). The outer and inner membranes were disrupted by peptides within 5 min. Thus, P5 and the truncated peptides killed bacteria by disrupting the bacteria membrane.

In conclusion, we investigated whether the truncated peptides of P5 retain antimicrobial activity. We confirmed that P5-CT1 and P5-NT1, which are truncated by two amino acids at the C-terminus or N-terminus, showed high antimicrobial activity against Gram-negative and Gram-positive bacteria, and no toxicity toward normal human cells compared with P5. The mechanism of action of the truncated peptides involved membrane disruption, the primary mechanism of AMPs. Therefore, as shorter peptides than their parent peptide, P5-CT1 and P5-NT1 would be less costly, and are suggested as potential therapeutic agents.

## Data Availability

The data set supporting the conclusions of this article is included within the article. Data and materials can also be requested from the corresponding author.
